# Minnelide Markedly Reduces Proteinuria in Mice with Adriamycin Nephropathy by Protecting Against Podocyte Injury

**DOI:** 10.1007/s12010-023-04333-z

**Published:** 2023-03-31

**Authors:** Baowei Ji, Junchao Liu, Yanli Ma, Ye Yin, Hong Xu, Qian Shen, Jian Yu

**Affiliations:** 1 Department of Nephrology, Children’s Hospital of Fudan University, Shanghai, China; 2 Department of Traditional Chinese Medicine, Children’s Hospital of Fudan University, Shanghai, China

**Keywords:** Adriamycin nephropathy,, Apoptosis,, Cytoskeleton,, Podocyte,, Triptolide

## Abstract

Minimal change disease (MCD) is the most common cause of idiopathic nephrotic syndrome in children. The current major therapy is hormones for most steroid-sensitive patients. However, many patients have recurrent relapses of the disease and require long-term immunosuppression, leading to significant morbidity due to the side effects of the drugs. Therefore, better drugs need to be urgently explored to treat nephrotic syndrome while avoiding the side effects of drugs. Minnelide, a water-soluble prodrug of triptolide, has been proved to be effective in treating cancers in many clinical trials. This study aimed to investigate the therapeutic effect of minnelide in mice with adriamycin (ADR) nephropathy, its underlying protection mechanisms, and its reproductive toxicity. Minnelide was administered intraperitoneally to 6–8-week female mice with adriamycin nephropathy for 2 weeks, and the urine, blood, and kidney tissues were taken to analyze the therapeutic effect. In addition, we evaluated reproductive toxicity by measuring the levels of gonadal hormones and observing the histological changes in ovaries and testes. Primary mouse podocytes were exposed to puromycin (PAN) to damage the cytoskeleton and induce apoptosis, and then, triptolide was used to evaluate the therapeutic effect and underlying protection mechanisms in vitro. It was observed that minnelide dramatically alleviated proteinuria and apoptosis in mice with adriamycin nephropathy. In vitro, triptolide ameliorated puromycin-induced cytoskeletal rearrangement and apoptosis via reactive oxygen species-mediated mitochondrial pathway. In addition, minnelide caused no reproductive toxicity to male and female mice. The results suggested that minnelide might be a promising drug for nephrotic syndrome.

## Introduction

Minimal change disease (MCD) is a common cause of idiopathic nephrotic syndrome (NS). In adults, it accounts for 10–15% of NS. In children, it accounts for up to 70–90% and is the most common pathological type of NS [48]. The incidence of MCD in children was reported to be about 2–7/100,000, and its main clinical manifestations are edema and intravascular insufficiency due to massive proteinuria. The effacement of foot process is the only morphological feature of MCD under electron microscopy. The etiology of MCD remains unclear, but immune dysregulation and podocyte modification are thought to play an important role in the pathogenesis [33, 48, 49].

Proteinuria is a common feature of NS, which results from the disruption of the glomerular filtration barrier. Podocytes are highly differentiated cells that play an important role in the glomerular filtration barrier. Podocyte injury is the main cause of proteinuria [1, 25]. Prednisone and immunosuppressants are the main drugs used to treat NS. However, in a large number of patients, the disease recurs repeatedly and requires long-term use of prednisone and immunosuppressants, with significant morbidity because of side effects [40]. Therefore, better drugs need to be urgently explored to treat NS while avoiding the side effects of drugs.

TWHF has been used in Chinese medicine for over 2000 years, mainly for the treatment of rheumatoid arthritis and other autoimmune diseases [11, 13]. Triptolide is one of the major active components of *Tripterygium wilfordii* Hook F (TWHF) [29]. Recent reports have shown that it has potent antitumor, immunosuppressive, and anti-inflammatory properties. Triptolide can have a significant therapeutic effect on tumors such as lung, liver, and pancreatic cancers, mainly through the induction of apoptosis [26, 56]. Triptolide not only induces apoptosis of immune cells and inflammation-related cells but also reduces the release of cytokines and pro-inflammatory mediators to exert anti-inflammatory and immunosuppressive effects [58]. Besides, triptolide can also effectively relieve proteinuria and reduce podocyte injury in vivo and in vitro [6, 50, 52, 57]. However, many causes, such as poor water solubility and bioavailability, and reproductive toxicity from long-term use, limit its clinical use [39].

The use of triptolide required its dissolution in the organic solvent dimethyl sulfoxide (DMSO), which was very harmful to mice or humans [47]. In addition, the oral administration of triptolide led to a decrease in its bioavailability, which has limited its clinical use [16, 39]. Therefore, the researchers have processed the triptolide to produce minnelide [8]. Minnelide is a water-soluble prodrug of triptolide, which has shown significant therapeutic effects against a large number of cancers in preclinical studies [2, 23, 35, 45]. In the body, minnelide is rapidly converted into triptolide in the presence of alkaline phosphatase [8]. Meanwhile, minnelide is currently in phase II clinical trials for treating pancreatic cancer. It is a promising drug that has gained a lot of attention. In addition, animal experiments and clinical trials have demonstrated that the hepatic, renal, and reproductive toxicity of triptolide is time- and dose-dependent [31, 38]. Therefore, we hypothesized that low-dose, short-term application of minnelide could effectively treat NS while avoiding reproductive toxicity, which will provide the experimental basis for the clinical treatment of NS with minnelide.

We established an ADR nephropathy mouse model of MCD in vivo to explore the therapeutic effect of minnelide on NS. In vitro, we used primary mouse podocytes to investigate the treatment effects of triptolide and the underlying protective mechanisms on PAN-induced podocyte injury. In addition, we also explored the reproductive toxicity of different doses of minnelide in female and male mice.

## Materials and Methods

### Drugs and Reagents

Minnelide (C_21_H_25_Na_2_O_6_P) was obtained from Shanghai SCR-Biotech Co., Ltd., Shanghai, China. The purity of minnelide was ≥ 98% by liquid chromatography-mass spectrometry. Triptolide (C_20_H_24_O6) was purchased from TCI Co., Shanghai, China. The purity of triptolide was > 98.0% by high-performance liquid chromatography. ADR (D515) was purchased from Sigma. The purity of ADR was 98.0–102.0% by high-performance liquid chromatography. PAN (A610593) was purchased from Sangong Bioengineering (Shanghai) Co., Ltd., Shanghai, China. The purity of PAN was ≥ 98.0%.

### Mouse Experiments

The ethics approval of animal experiments was granted by the Ethics Committee of Fudan University (approval number IDM2021055). For the ADR nephrology mouse experiment, 15 C57BL/6 female mice (6–8 weeks) were randomly assigned to the control group, ADR group, and minnelide + ADR group. The mice in the ADR and minnelide + ADR groups received ADR (25 mg/kg) on day 0 in the tail vein, while the mice in the control group received the same dose of saline in the tail vein. The mice in the minnelide + ADR group received an intraperitoneal injection of minnelide [200 μg/kg/d] from day 1 to day 14, while the mice in the control and ADR groups received an identical volume of saline intraperitoneally.

For evaluating the reproductive toxicity of minnelide, 20 C57BL/6 male mice (6–8 weeks) and 20 C57BL/6 female mice (6–8 weeks) were randomly divided into four groups. The mice in the control group were intraperitoneally injected with the same dose of normal saline for 2 weeks. Mice were injected intraperitoneally with 100 μg/kg/d of minnelide for 2 weeks in the 100 μg/kg/d group, 200 μg/kg/d of minnelide for 2 weeks in the 200 μg/kg/d group, and 400 μg/kg/d of minnelide for 2 weeks in the 400 μg/kg/d group.

### Tissue Preparation and Blood Collection

For the ADR nephrology mouse experiment, the urine was collected for 24 h using a metabolic cage at 2 weeks and then centrifuged at 12,000 rpm for 15 min. The supernatant was aspirated and stored at – 80 °C. Blood was drawn from mice via the jaw vein at 300 μL each and then centrifuged at 12,000 rpm for 15 min. The supernatant was aspirated and stored frozen at – 80 °C. The urine microalbumin and creatinine levels were measured using a microalbumin assay kit and a urine creatinine assay kit. The serum albumin, triglyceride, and cholesterol levels were measured using assay kits. All assay kits were obtained from the Nanjing Jiancheng Bioengineering Institute, Nanjing, China.

For evaluating the reproductive toxicity of minnelide, the ovaries and testes were removed from female and male mice separately after 2 weeks and then embedded in paraffin. Subsequently, the tissues were stained with hematoxylin and eosin for histological studies. For female mice, the process of isolating the supernatant of blood was the same as earlier. The serum steroid and gonadotrophin levels were measured using assay kits (Nanjing Jiancheng Bioengineering Institute).

### Transmission Electron Microscopy

The renal cortices were cut to a size of 1 mm^3^ and then rinsed three times in phosphate buffer (pH 7.4) for 15 min each. They were then dehydrated with various concentrations of alcohol for 20 min each and 100% acetone two times for 15 min each. Using acetone permeation embedding, the ultra-thin sections were cut into 60- to 80-mm slices, stained, and then photographed under a transmission electron microscope (Hitachi HT7700).

### Immunofluorescence Labeling

The paraffin sections of kidney tissues were dewaxed with water and then antigenically repaired using antigen repair solution (pH 8.0). The sections were shaken dry and then blocked with serum. They were incubated overnight at 4 °C with podocin rabbit polyclonal antibody (Proteintech, Wuhan, China) and cd2ap rabbit polyclonal antibody (Sigma, NY, USA), followed by secondary antibody, stained with 4′,6-diamidino-2-phenylindole (DAPI), and finally blocked and photographed under an Olympus FV3000 laser confocal microscope (Olympus, Japan).

### Western Blot Analysis

The proteins were extracted from kidney tissues or cells using RIPA lysate, protein extraction from cytoplasm, and mitochondria was performed according to the Cell Mitochondria Isolation Kit (Shanghai Biyuntian Biotechnology Co., Ltd., Shanghai, China). Then, proteins were electrophoresed with 10% sodium dodecyl sulfate–polyacrylamide gel electrophoresis (SDS-PAGE), transferred to membranes, and incubated with 5% skimmed milk at room temperature for 1 h. The membrane was washed with TBST for 10 min × 3. Then, primary antibodies anti-cleaved-caspase-3 rabbit pAb (CST, USA), anti-cytochrome c rabbit pAb (Servicebio, China), anti-Bax Ab (ab216494), and anti-Bcl-2 Ab (ab194583) were added separately and overnight for 4 °C. The membrane was washed with TBST for 10 min × 3, and the secondary antibodies (Servicebio, China) were added. The membrane was washed with TBST for 10 min × 3 and exposed to light. The protein quantitative analysis was performed using ImageJ software.

### Podocyte Culture and Treatment

Primary mouse podocytes were isolated as described in our previous protocol [9]. Briefly, the kidney cortex of the mice was removed and minced, and then, the glomeruli were isolated using a three-step sieve under aseptic conditions. After 6 days of primary culture, the glomeruli were removed and the podocytes were digested with trypsin and then replated onto plates, followed by the relevant cell experiments. Primary mouse podocytes were cultured in a 1:1 mixed KI medium (DMEM/F12 medium containing HEPES 10 mmol/L, ITS-X (with transferrin 0.55 g/L, sodium selenite 67 mg/L, insulin 1 g/L), penicillin 10^5^ U/L, streptomycin 100 mg/L), and 3T3 medium (DMEM medium–high glucose containing 200 g/L fetal bovine serum, HEPES 10 mmol/L, sodium glutamate 2 mmol/L, sodium pyruvate 2 mmol/L, penicillin 10^5^ U/L, streptomycin 100 mg/L) at 37 °C. Primary mouse podocytes were planted in 10-cm cell culture dishes, and they were divided into three groups: control group, PAN group, and triptolide pretreatment group. In the PAN group, the podocyte injury was induced by treatment with 50 μg/mL PAN for 24 h. In the triptolide pretreatment group, the podocytes were pretreated with 10 ng/mL triptolide for 30 min before exposure with PAN for 24 h.

### CCK8 Assay

The drug concentration of triptolide was assessed using cell counting kit-8 (CCK-8) assay (APExBIO Technology LLC, TX, USA). Briefly, the cells were resuspended and then inoculated overnight in 96-well plates, followed by dropwise addition of various concentrations of triptolide. The OD values were measured after 24 h. Data are presented as means ± standard deviation (SD) of three experiments.

### Detection of Apoptosis

PE–annexin-V and terminal deoxynucleotidyl transferase dUTP nick end labeling (TUNEL) assay were used to detect apoptosis. PE–annexin-V apoptosis detection kit and one-step TUNEL apoptosis assay kit were purchased from Shanghai Biyuntian Biotechnology Co., Ltd., Shanghai, China. All methods were performed following the manufacturer’s protocol. Briefly, 10,000 different groups of cells were measured using flow cytometry analysis. TUNEL-stained images were observed under a fluorescence microscope (Olympus, Japan), and three images from each group were analyzed.

### Measurement of ROS Levels in Renal Tissues and Podocytes

For renal tissues, the frozen sections of kidney tissue were rewarmed at room temperature and then incubated with reactive oxygen species (ROS) staining solution (Sigma, USA) at 37 °C for 30 min protected from light. The sections were then incubated with DAPI for 30 min at room temperature protected from light, washed three times with phosphate-buffered saline (PBS), and then observed under an Olympus FV3000 laser confocal microscope (Olympus).

The intracellular ROS were detected following the instructions of a reactive oxygen species assay kit (Shanghai Biyuntian Biotechnology Co., Ltd., Shanghai, China). Briefly, podocytes were inoculated in a six-well plate, left to adhere, and then intervened using DCFH-DA, followed by the stimulation of podocytes with 50 μg/mL puromycin for 30 min, and finally observed under the Olympus FV3000 laser confocal microscope (Olympus).

### Measurement of Mitochondrial Membrane Potential

The mitochondrial membrane potential was detected following the instructions of an enhanced mitochondrial membrane potential assay kit (Shanghai Biyuntian Biotechnology Co., Ltd.) with JC-1. Briefly, the cells were inoculated on six-well plates overnight. After the cells were plastered, the culture fluid was aspirated, the cells were washed once with PBS, and then 1 mL of the cell culture fluid was added. Immediately afterward, 1 mL of JC-1 staining working solution was added and mixed thoroughly. The cells were incubated for 20 min at 37 °C in a cell incubator. At the end of incubation, the supernatant was aspirated and washed twice with JC-1 staining buffer. Then, 2 mL of the cell culture solution was added, and finally, the cells were observed under the Olympus FV3000 laser confocal microscope (Olympus).

### Measurement of Mitochondrial Morphology

The mitochondrial morphology was detected following the instructions of Mito-Tracker Red CMXRos (Shanghai Biyuntian Biotechnology Co., Ltd.). Briefly, the cells were resuspended in six-well plates overnight. After the cells were plastered, the cell culture medium was removed, the prepared Mito-Tracker Red CMXRos working solution was added, and the cells were incubated at 37 °C for 30 min. Subsequently, the Mito-Tracker Red CMXRos working solution was removed, fresh cell culture pre-warmed at 37 °C was added, and finally, the cells were observed under the Olympus FV3000 laser confocal microscope (Olympus).

### Statistical Analysis

Statistical analyses were performed with SPSS (version 20.0) and GraphPad Prism (version 9.0). The results were expressed as mean ± SD. One-way analysis of variance was used for comparison between groups. Statistical significance was set at *P* < 0.05.

## Results

### Minnelide Improved the Body Weight and General State of Mice with Adriamycin Nephropathy

**Fig. 1:**
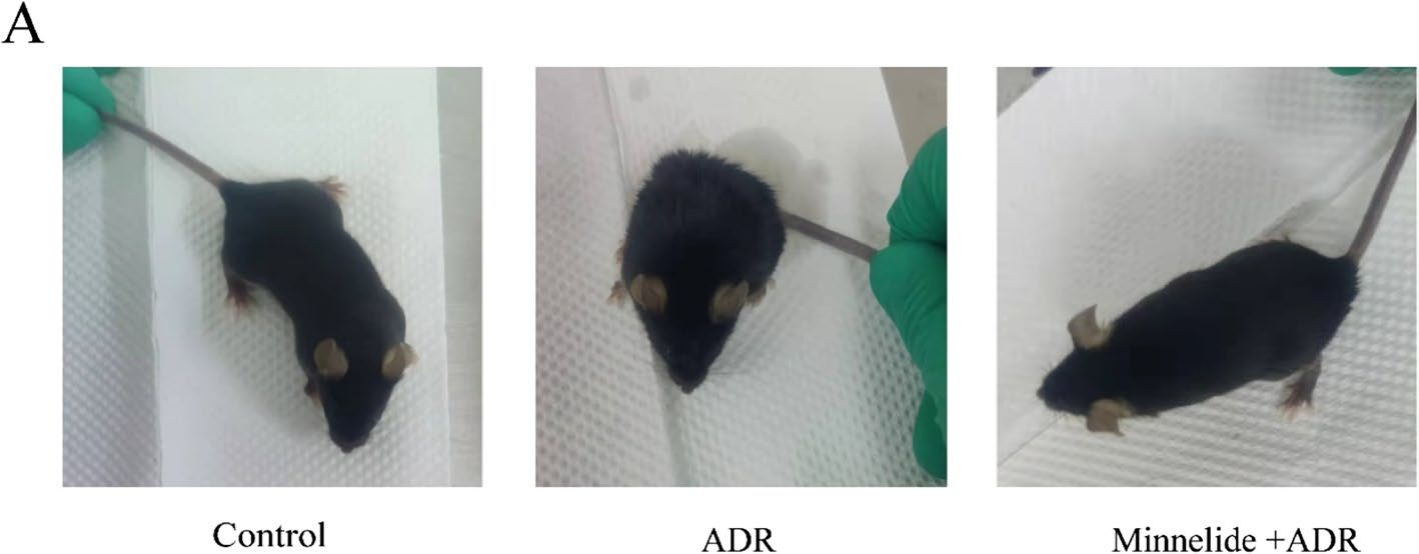
Minnelide improved the general state of mice with adriamycin nephropathy

As shown in Table 1, the body weight significantly decreased in the ADR group and increased with minnelide therapy. Also, the mice in the ADR group appeared to have sparser hair, significant loss of mobility, and curly body appearance. The mice in the minnelide treatment group appeared to be significantly stronger than the mice in the ADR group (Fig. 1A).

**Table 1:** The effect of minnelide on body weight in mice with adriamycin nephropathy (mg)

	0 week	1 week	2 weeks
Control	19.92 ± 0.51	19.82 ± 1.04	19.55 ± 1.27
ADR	20.18 ± 1.88	14.86 ± 0.76^****^	13.79 ± 0.59^****^
Minnelide + ADR	20.00 ± 0.48	16.44 ± 0.76^#^	16.76 ± 0.55^###^

Data were represented as mean ± SD; *n* = 5 per group. ^****^P < 0.0001 versus Control groups. ^#^*P* < 0.05 and ^###^*P* < 0.001 versus ADR groups

### Renal Protective Effects of Minnelide for Mice with Adriamycin Nephropathy

**Fig. 2:**
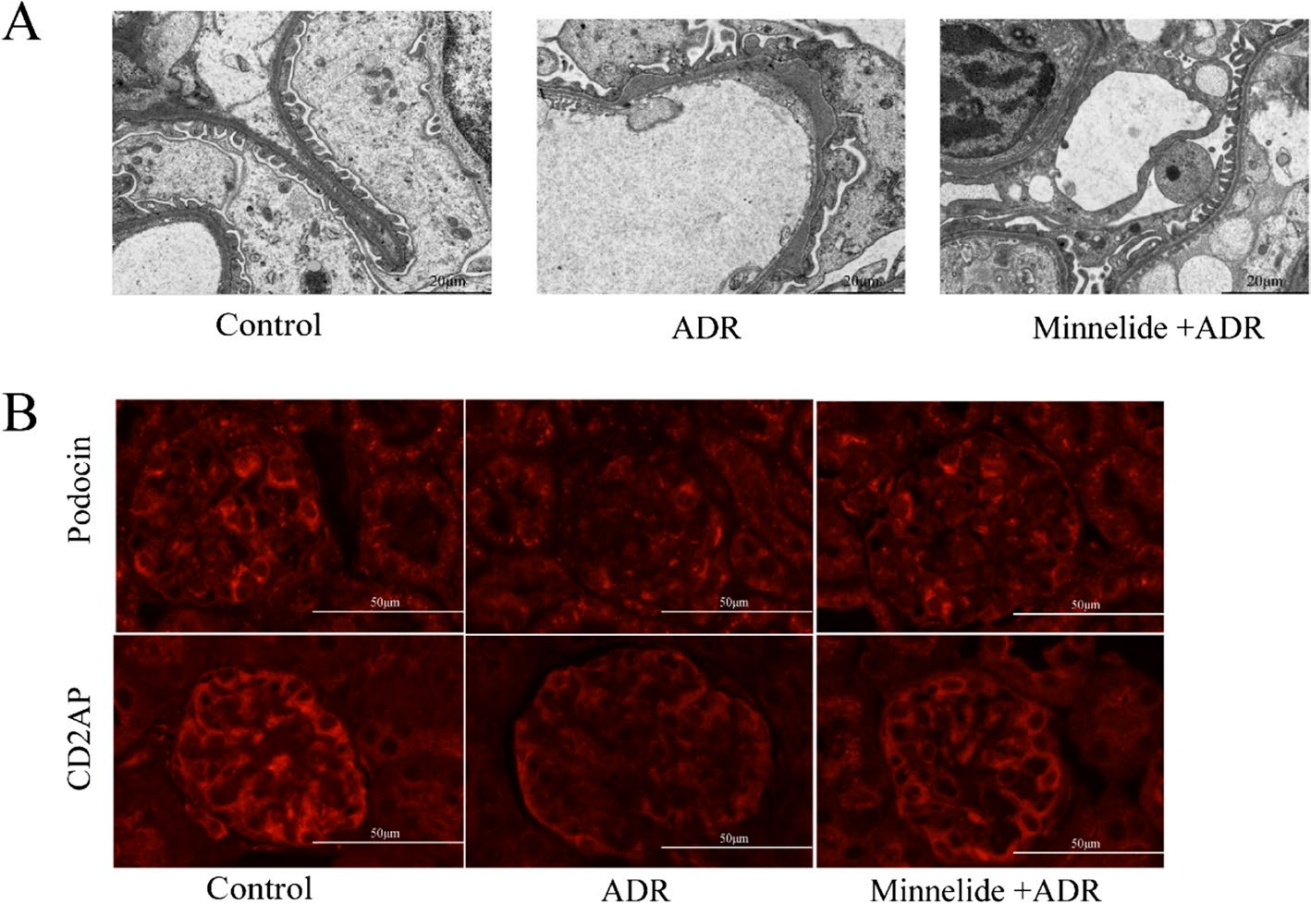
Renal protective effects of minnelide in mice with adriamycin nephropathy. **A** Transmission electron microscopy, bar = 2.0 μm. **B** Effect of minnelide on podocin and cd2ap expression and distribution in mice. Original magnification × 400

The urine albumin/creatinine ratios reduced markedly in the minnelide treatment group compared with the ADR group (*P* < 0.0001; Table 2). Th serum albumin levels significantly increased in the minnelide treatment group compared with the ADR group (*P* < 0.001; Table 2). After 2 weeks of minnelide treatment, both serum triglyceride and cholesterol levels significantly reduced in the minnelide treatment group compared with the ADR group (*P* < 0.0001, *P* < 0.001; Table 2). Transmission electron microscopy revealed lighter effacement of the podocyte foot processes in the minnelide treatment group compared with the ADR group (Fig. 2A). Meanwhile, the immunofluorescence results showed that the expression of podocin and cd2ap levels significantly increased in the minnelide treatment group compared with the ADR group (Fig. 2B). Collectively, these data indicated a protective effect of minnelide on mice with adriamycin nephropathy.

**Table 2:** The effect of minnelide on blood and urinary indicators in mice with adriamycin nephropathy

	Urine albumin to creatinine (μg/mg)	Albumin (g/L)	Triglyceride (mmol/L)	Cholesterol (mmol/L)
Control	39.38 ± 8.58	21.26 ± 0.69	1.28 ± 0.16	2.52 ± 0.05
ADR	150.0 ± 19.31^****^	15.31 ± 0.44^****^	2.61 ± 0.24^****^	3.46 ± 0.18^****^
Minnelide + ADR	58.52 ± 3.67^####^	17.75 ± 0.75^###^	1.76 ± 0.07^####^	2.86 ± 0.22^###^

Data were represented as mean ± SD; *n* = 5 per group

^****^P < 0.0001 versus control groups

^###^*P* < 0.001 and ^####^*P* < 0.0001 versus ADR groups

### Minnelide Alleviated Apoptosis in Mice with Adriamycin Nephropathy

**Fig. 3:**
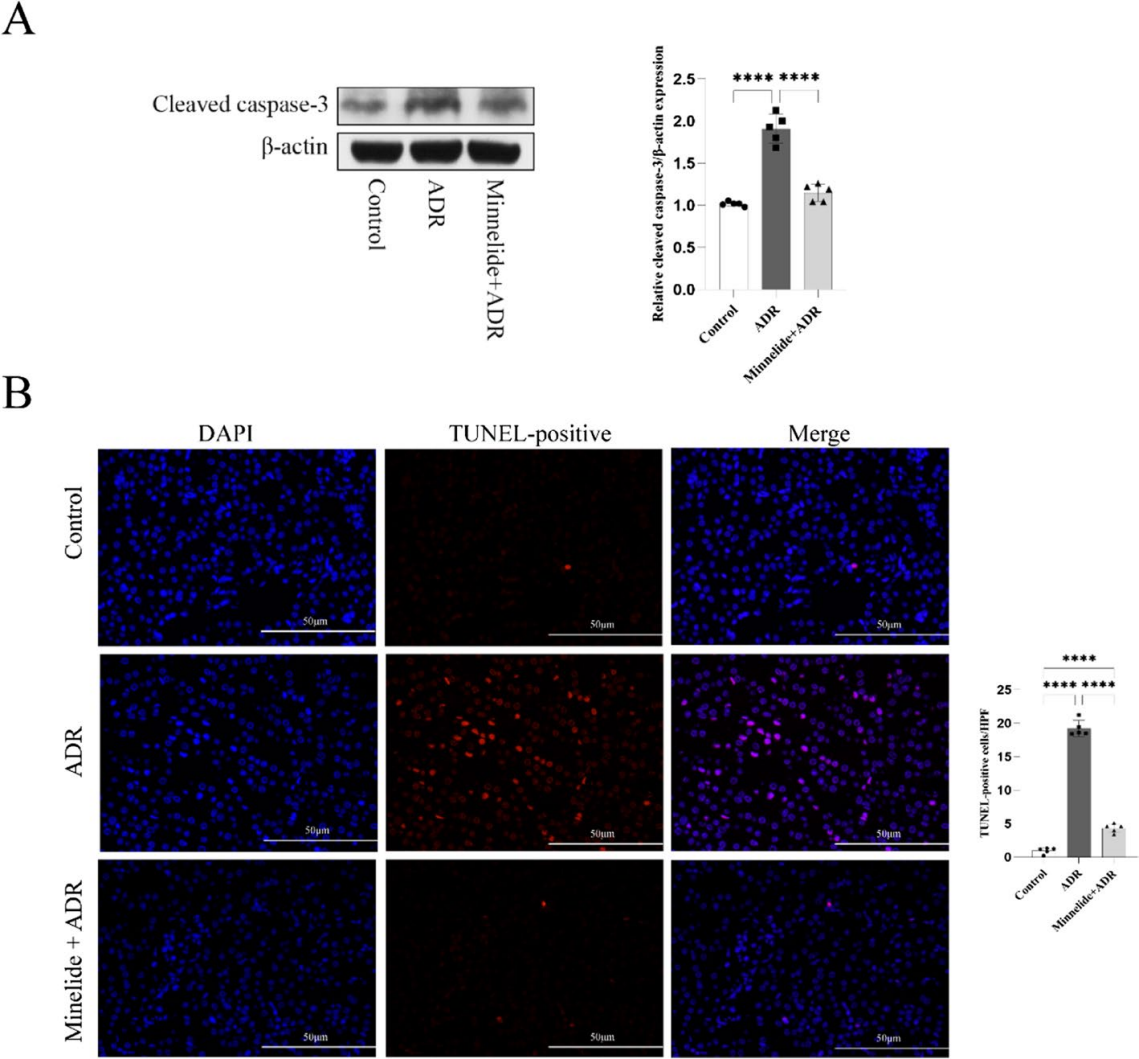
Minnelide improved ADR-induced renal cell apoptosis. **A** Protein level of cleaved caspase-3 in kidneys from each group. **B** Images of TUNEL staining from each group. The red color denotes apoptotic cells, and the blue color denotes DAPI-stained cells; *n* = 5 per group. Comparisons between groups were assessed using a one-way analysis of variance. Data were represented as mean ± SD. ^****^P < 0.0001. Original magnification × 400

Western blot analysis showed that the expression of apoptosis-related protein cleaved caspase-3 was increased in mice with ADR-induced nephrology. Minnelide therapy could dramatically reduce the level of cleaved caspase-3, demonstrating that minnelide could alleviate apoptosis in ADR-induced kidneys (*P* < 0.0001; Fig. 3A). TUNEL staining was used to show the extent of renal cell apoptosis. The minnelide treatment group showed a lower number of apoptotic cells compared with the ADR group (*P* < 0.0001; Fig. 3B).

### Triptolide Prevented PAN-Induced Podocyte Cytoskeletal Injury and Apoptosis

**Fig. 4:**
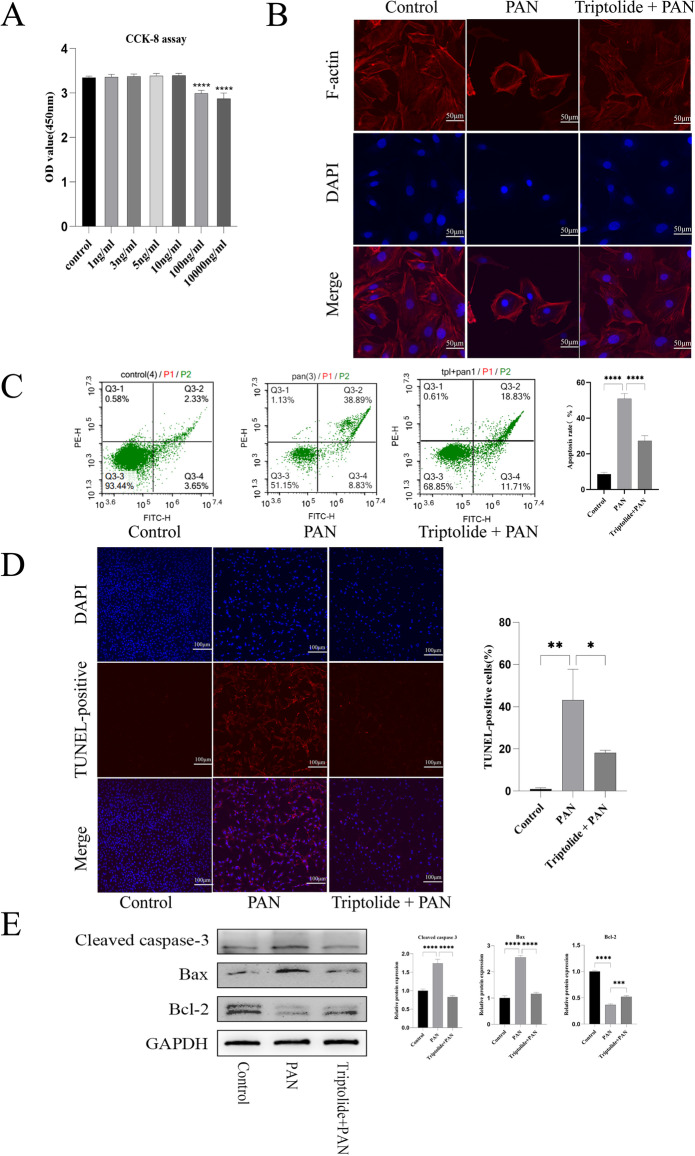
Triptolide prevented PAN-induced podocyte cytoskeletal injury and apoptosis. **A** CCK-8 assays. **B** Triptolide pretreatment prevented PAN-induced cytoskeleton disorganization. Red fluorochrome indicates F-actin, and blue indicates the nucleus. Original magnification × 400. **C** PE–annexin-V staining followed by FCM demonstrated that PAN-induced apoptosis was alleviated by triptolide. **D **TUNEL assays of the podocytes treated as indicated. Quantification of the results is shown on the right panel. Original magnification × 200. **E** Protein level of Bax, Bcl-2, and cleaved caspase-3 in podocytes from each group. Comparisons between groups were assessed using a one-way analysis of variance. Data were represented as mean ± SD. ^*^P < 0.05, ^**^P < 0.01,^****^P < 0.0001. (based on three triplicate tests)

We used the PAN injury model of cultured podocytes pretreated with triptolide to explore whether triptolide had a protective effect against PAN-induced podocyte injury. First, the CCK-8 assay showed that podocyte proliferation was not affected by drug concentration less than or equal to 10 ng/mL. Therefore, 10 ng/mL was selected as the concentration for in vitro experiments (Fig. 4A). We observed using phylloidin staining that PAN-induced podocyte cytoskeletal injury was shown by the disorganization of F-actin stress fibers compared with control cells. However, pretreatment with 10 ng/mL triptolide recovered the number of F-actin stress fibers and their structure prominently (Fig. 4B). TUNEL assays and PE–annexin-V staining indicated that 10 ng/mL triptolide pretreatment could significantly alleviate podocyte apoptosis compared with the PAN group (Fig. 4C, D). We further examined the expression of apoptosis-related proteins, such as Bax, Bcl-2, and cleaved caspase-3. The results demonstrated that puromycin significantly increased the expression of the pro-apoptotic protein Bax and decreased the expression of the inhibitory protein Bcl-2 compared with the control group. In addition, the expression of cleaved caspase-3 also significantly increased, while treatment with triptolide improved the expression of these proteins (Fig. 4E).

### Minnelide and Triptolide Attenuated ROS Production In Vivo and In Vitro

**Fig. 5:**
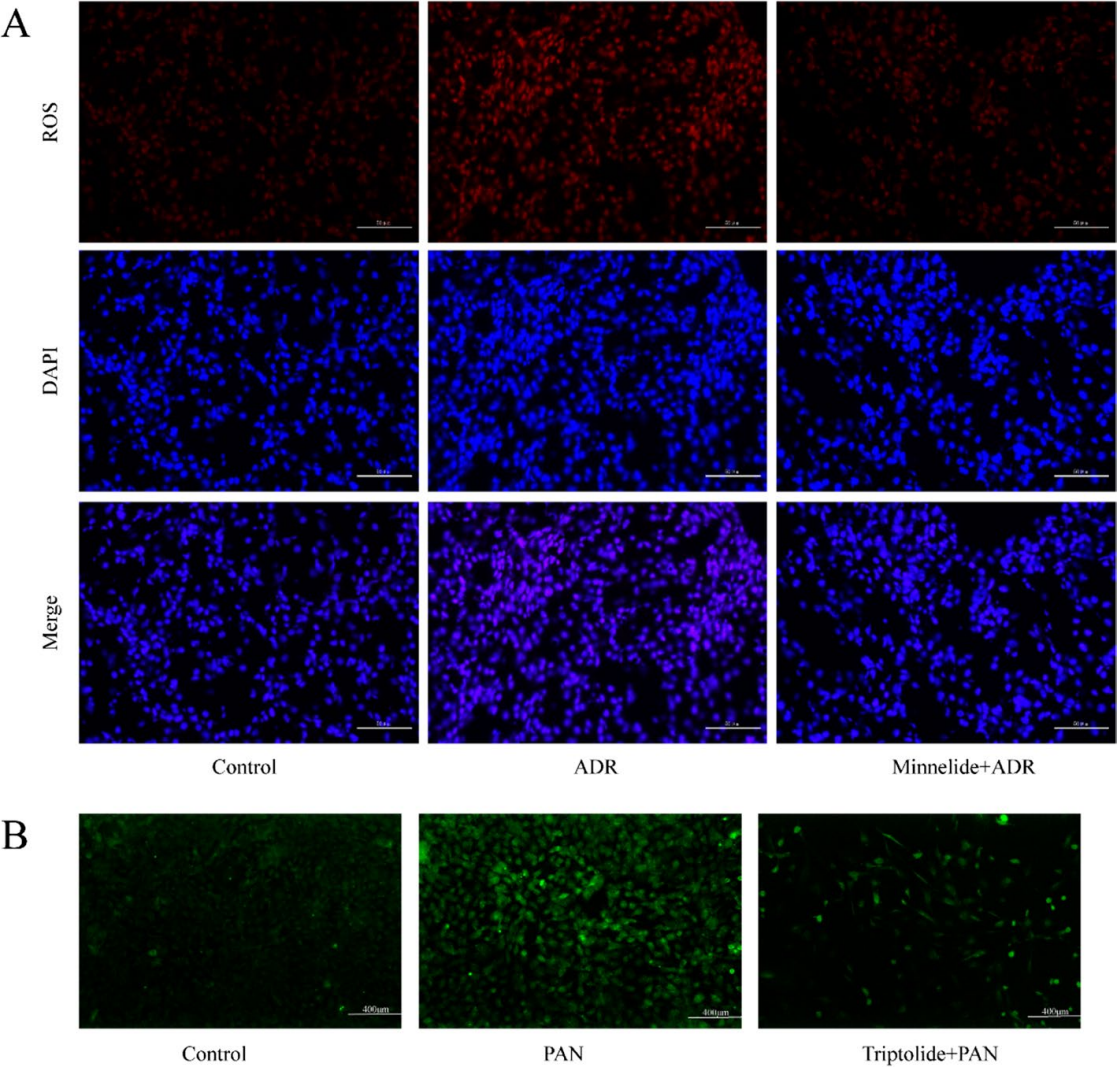
Minnelide and triptolide could attenuate ROS levels in vivo and in vitro. **A** Levels of ROS in renal tissues were detected by ROS staining. Original magnification × 400. **B** Levels of ROS in podocytes were detected using the ROS assay kit. Original magnification × 100

The ROS levels were closely related to apoptosis. As shown in Fig. 5A, the ROS levels in the ADR group increased, and minnelide therapy could reduce ROS levels significantly in vivo. Similarly, the increased ROS levels in podocytes induced by PAN improved after triptolide pretreatment (Fig. 5B).

### Triptolide Protected Podocytes by Inhibiting the Mitochondrial Apoptosis Pathway

**Fig. 6:**
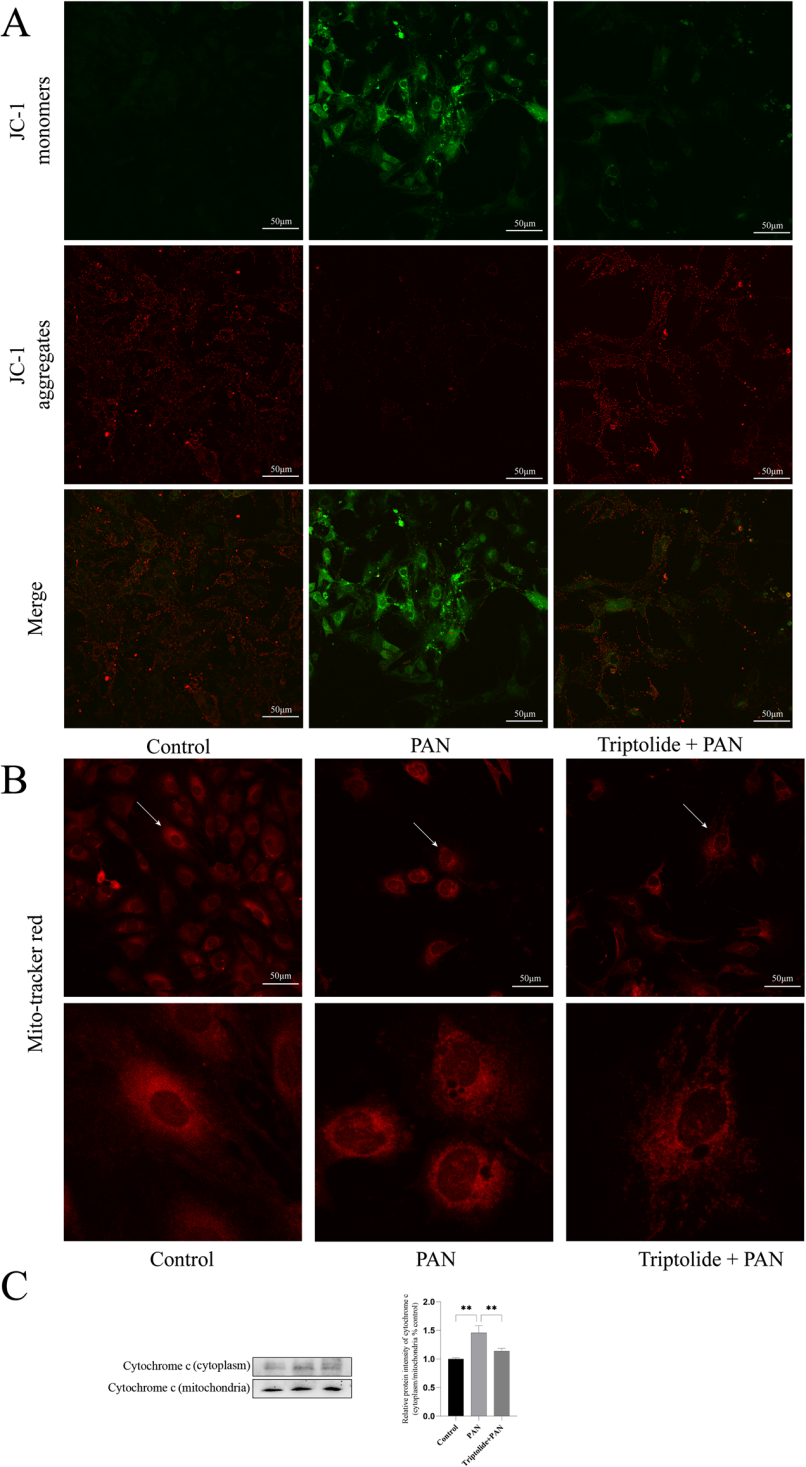
Triptolide protected podocytes by inhibiting the mitochondrial apoptosis pathway. **A** Mitochondrial membrane potential assay kit with JC-1 detected the mitochondrial membrane potential of each group of podocytes. Original magnification × 400. **B** Mito-Tracker Red CMXRos detected the mitochondrial morphology of each group of podocytes. Original magnification × 400. **C** Protein level of cytochrome c in podocytes from each group. **P < 0.01. (based on three triplicate tests)

Mitochondria are the main site of ROS production. Elevated ROS levels can cause mitochondrial dysfunction and thus induce apoptosis. Therefore, we investigated whether triptolide could protect the functioning of mitochondria. The mitochondrial membrane potential of podocytes decreased significantly after 24 h of puromycin treatment, and JC-1 formed green fluorescence. The pretreatment with triptolide significantly increased the mitochondrial membrane potential, and JC-1 turned into a red aggregates (Fig. 6A). Next, we observed whether triptolide could protect the morphology of mitochondria in podocytes. After 24 h of puromycin treatment, the number of fragmented mitochondria in podocytes increased compared with the control group, and triptolide pretreatment significantly reduced the fragmentation of mitochondria in podocytes (Fig. 6B). Several studies reported that mitochondrial damage promoted the release of cytochrome c from mitochondria into the cytoplasm. Next, we examined the protein expression of cytochrome c in the mitochondria and cytoplasm by Western blot analysis. The results showed that puromycin significantly increased the protein expression of cytochrome c in cytoplasm compared with the control group, while triptolide pretreatment significantly decreased its expression (Fig. 6C).

### Reproductive Toxicity of Minnelide in Mice

**Fig. 7:**
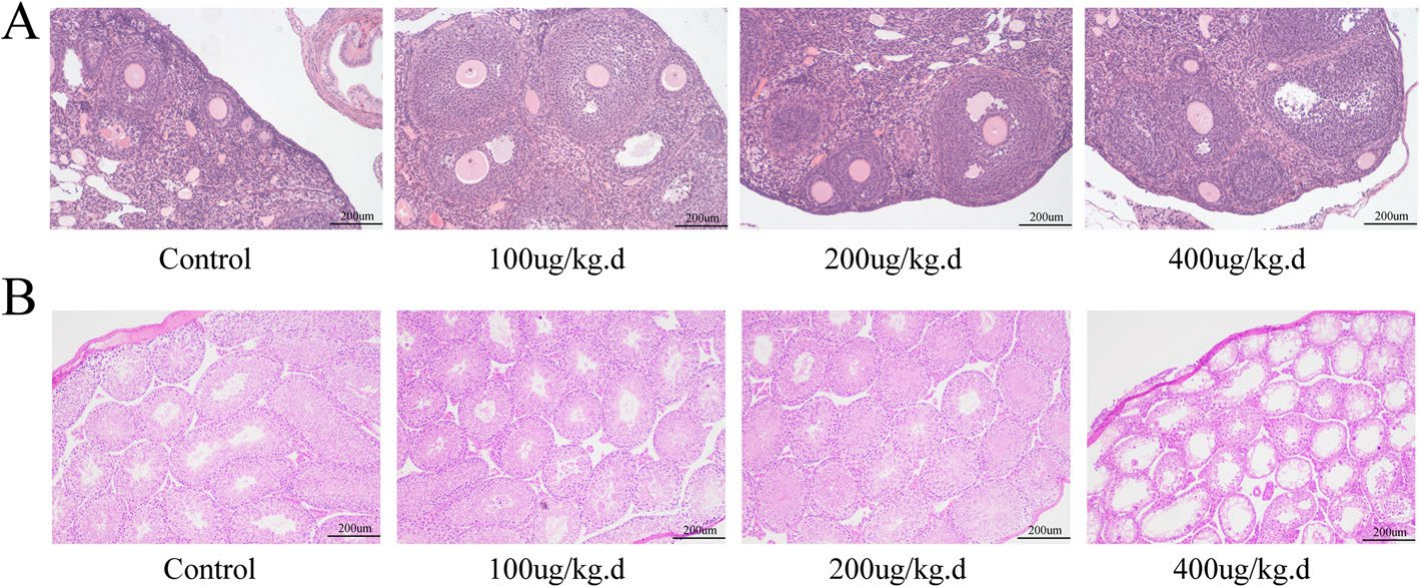
Effects of minnelide on ovarian histology in female mice and on testicular histology in male mice. **A** Ovary. **B** Histopathological changes in the testes. Original magnification × 100

We injected female and male mice with different doses of minnelide for 2 weeks to investigate the reproductive toxicity of minnelide. For female mice, as shown in Table 3, the dose of 400 μg/kg/d of minnelide significantly increased the FSH and LH levels and reduced the E2 level, compared with the control group. However, the low doses did not influence the FSH, LH, P, and E2 levels. The histology of the ovarian follicles suggested that different doses of minnelide had no obvious effect on follicular development in treated ovaries (Fig. 7A). For male mice, germ cell degeneration, exfoliation, and vesicles of varying sizes were found in the 400 μg/kg/d group. However, both 100 and 200 μg/kg/d caused no obvious impairments in testes (Fig. 7B).

**Table 3:** The effect of minnelide on serum-steroid and gonadotropin in female mice

	P (ng/mL)	E2 (pg/mL)	FSH (mIU/mL)	LH (mIU/mL)
Control	22.59 ± 4.05	67.24 ± 9.36	86.73 ± 18.35	28.73 ± 2.30
100 μg/kg day	23.65 ± 2.06	51.21 ± 7.18	85.62 ± 24.19	30.28 ± 4.81
200 μg/kg day	17.30 ± 4.11	50.36 ± 6.30	75.55 ± 13.60	28.92 ± 6.09
400 μg/kg day	15.06 ± 4.06	43.30 ± 5.46^**^	126.0 ± 10.75^*^	34.54 ± 4.75^*^

P, progesterone; E2, estradiol; FSH, follicle-stimulating hormone; LH, luteotropic hormone. Data were represented as mean ± SD; *n* = 5 per group. ^*^P < 0.05 and ^**^P < 0.01 versus control groups

## Discussion

MCD is common in NS and is characterized by massive proteinuria, hypoalbuminemia, edema, and hyperlipidemia [48]. At present, glucocorticoids and immunosuppressants are the main treatments for NS [32]. However, some patients presenting with recurrent relapses required the long-term use of drugs to control the condition, the side effects of the long-term use of glucocorticoids and immunosuppressants cannot be ignored [12]. In addition, if proteinuria persists and kidney function continues to decline, it will eventually lead to end-stage renal disease [51], and hence, better drugs are urgently needed to treat NS.

Triptolide is effective in treating glomerulonephritis, and showed dramatic effects in terms of decreasing proteinuria [30, 43]. For PAN-induced nephrology, triptolide could significantly reduce proteinuria in both pretreated and treated mice [57]. In addition, Chen et al. [6] created an animal model of membranous nephropathy. The treatment with triptolide could significantly reduce proteinuria in mice. Gao et al. [17] also found that triptolide markedly reduced proteinuria and protected renal structure and function in db/db mice with diabetes. For Henoch–Schönlein purpura nephritis (HSPN), a controlled clinical trial showed that triptolide could markedly relieve the symptoms of HSPN in children [52]. However, the use of triptolide in vivo has remained a challenge because of limited solubility and reproductive toxicity from the long-term use [5, 39, 47]. The injection of ADR into mice could produce proteinuria and pathological lesions of MCD [24]. In this study, our results showed that minnelide could reduce ADR-induced proteinuria and prevent ADR-induced podocyte apoptosis in vivo. Meanwhile, our findings also indicated that minnelide at 200 μg/kg/d for 14 days showed no reproductive toxicity in both female and male mice. Therefore, minnelide might be a promising drug for NS.

The podocyte is a terminally differentiated cell located in the outer layer of the glomerular basement membrane [42]. Podocyte injury is the core link in the occurrence and development of proteinuria and various glomerular diseases [27, 37]. Previous studies demonstrated that triptolide could alleviate podocyte injury. Zheng et al. [57] found that triptolide could reduce PAN-induced podocyte cytoskeletal injury. In addition, Wang et al. [50] found that triptolide could attenuate podocyte apoptosis in metronidazole-induced injury in zebrafish and PAN-treated podocytes. PAN could injure podocytes, leading to actin cytoskeleton rearrangement and podocyte apoptosis [20, 36]. In our study, we found that minnelide could alleviate apoptosis in mice with adriamycin nephropathy. In vitro, pretreatment with triptolide could also alleviate PAN-induced cytoskeleton disorganization and podocyte apoptosis.

The increase in ROS production in podocytes contributed to mitochondrial oxidative stress and podocyte apoptosis [18, 59]. Several exposures such as high glucose concentrations, PAN, and ADR could increase the production of ROS [21, 34, 41]. Previous studies indicated that triptolide could reduce the level of ROS in vitro. Zheng et al. [57] found that triptolide treatment significantly attenuated the increase in puromycin-induced ROS levels in podocytes. Yang et al. [54] also found that triptolide could reduce ROS levels in 266–6 cells exposed to caerulein. Gao et al. [15] also found that triptolide alleviated oxidative stress in podocytes induced by high glucose concentrations. In our study, we found that minnelide treatment could reduce the ROS levels in mice with adriamycin nephropathy. In vitro, triptolide pretreatment could also reduce the ROS levels in podocytes exposed to PAN.

Some recent studies reported that mitochondria were central to the production of ROS and the increased production of ROS could cause mitochondrial dysfunction and programmed cell death in cells [22, 46]. The elevated ROS levels in mitochondria led to the changes in mitochondrial membrane potential [14, 59], and the decline in mitochondrial membrane potential was a hallmark event in the early stages of apoptosis [3, 28]. Previous studies showed that the damage to mitochondria could increase the expression of the pro-apoptotic related protein Bax and decrease the expression of the anti-apoptotic protein Bcl-2, which controlled the permeability of the outer mitochondrial membrane and allowed the release of cytochrome c [3, 4, 55]. The released cytochrome c could bind to the adaptor protein Apaf-1, leading to the activation of caspases [10, 44]. In our study, we found that puromycin led to the loss of mitochondrial membrane potential, increased expression of apoptosis-related proteins such as Bax, decreased Bcl-2 expression, and increased cytochrome c release, resulting in increased cleaved caspase-3 expression. Pretreatment with triptolide significantly ameliorated these changes. Therefore, triptolide might protect podocytes via ROS-mediated mitochondrial pathway.

Reproductive toxicity has been a serious obstacle to the clinical use of triptolide [7, 53]. The dose- and time-dependent reproductive toxicity of triptolide was reported in both female and male rats [7]. For males, the long-term treatment (8 weeks) with triptolide produced significant changes in seminiferous tubules and epididymis [38]. For reproductive toxicity in female mice, the long-term treatment (90 days) with triptolide prolonged the cycle and reduced estrogen levels and the weights of ovaries and uterus [31]. Therefore, we investigated the reproductive toxicity of minnelide in both female and male mice. For female mice, the development of follicles in the ovaries was not significantly affected after 2 weeks of treatment; both 100 and 200 μg/kg/d had no significant effect on gonadal hormones. For male mice, 400 μg/kg/d could increase germ cell degeneration and exfoliation in testes, but both 100 and 200 μg/kg/d had no significant effect on testes after treatment with minnelide for 2 weeks. Previous studies demonstrated that 200 μg/kg/d minnelide treatment for 5 weeks had no significant effect on the body weight of mice [45], and 0.21 μg/kg/d minnelide treatment for 2 weeks did not affect the blood indexes of mice [19]. Meanwhile, Minneamrita Therapeutics LLC initiated many clinical trials (NCT01927965, NCT03117920, NCT03129139, and NCT03760523) for minnelide [47]. Collectively, these data demonstrated that treatment with 200 μg/kg/d for 2 weeks was relatively safe.

In conclusion, minnelide could significantly reduce proteinuria and protect renal structure and function in mice with adriamycin nephropathy. The results were presented as an improvement in podocyte foot process effacement, restoration of podocin and cd2ap distribution, and alleviation of ADR-induced apoptosis. In vitro, triptolide protected podocytes from PAN-induced cytoskeleton rearrangements and apoptosis via ROS-mediated mitochondrial pathway. Also, short-term and low doses of minnelide were relatively safe for female and male mice.

## Conclusion

Our study demonstrated that minnelide had a prominent antiproteinuric effect on ADR-induced nephrology in vivo and reduced PAN-induced podocyte injury in vitro. Meanwhile, no obvious reproductive toxicity was reported after intraperitoneal injection of minnelide for 2 weeks. Therefore, minnelide might be a promising drug for NS.

## Data Availability

Not applicable.
